# Effect of Sc and Zr Additions on Recrystallization Behavior and Intergranular Corrosion Resistance of Al-Zn-Mg-Cu Alloys

**DOI:** 10.3390/ma14195516

**Published:** 2021-09-23

**Authors:** Peng Xia, Shuncheng Wang, Huilan Huang, Nan Zhou, Dongfu Song, Yiwang Jia

**Affiliations:** 1 Institute of New Materials, Guangdong Academy of Sciences, Guangzhou 510650, China; xiapeng1987xp@163.com (P.X.); hlhuang@gimp.gd.cn (H.H.); zhounan@gimp.gd.cn (N.Z.); songdongfu@gimp.gd.cn (D.S.); ywjia@gimp.gd.cn (Y.J.); 2 Guangdong Provincial Key Laboratory of Metal Toughening Technology and Application, Institute of New Materials, Guangdong Academy of Sciences, Guangzhou 510650, China; 3 Guangdong Xingfa Aluminum Company Limited, Foshan 528137, China

**Keywords:** Al-Zn-Mg-Cu alloy, recrystallization, intergranular corrosion, scandium

## Abstract

The recrystallization and intergranular corrosion behaviors impacted by the additions of Sc and Zr in Al-Zn-Mg-Cu alloys are investigated. The stronger effect of coherent Al_3_(Sc_1−x_Zr_x_) phases on pinning dislocation resulted in a lower degree of recrystallization in Al-Zn-Mg-Cu-Sc-Zr alloy, while the subgrain boundaries can escape from the pinning of Al_3_Zr phases and merge with each other, bringing about a higher degree of recrystallization in Al-Zn-Mg-Cu-Zr alloy. A low degree of recrystallization promotes the precipitation of grain boundary precipitates (GBPs) with a discontinuous distribution, contributing to the high corrosion resistance of Al-Zn-Mg-Cu-Sc-Zr alloy in the central layer. The primary Al_3_(Sc_1−x_Zr_x_) phase promotes recrystallization due to particle-stimulated nucleation (PSN), and acts as the cathode to stimulate an accelerated electrochemical process between the primary Al_3_(Sc_1−x_Zr_x_) particles and GBPs, resulting in a sharp decrease of the corrosion resistance in the surface layer of Al-Zn-Mg-Cu-Sc-Zr alloy.

## 1. Introduction

Al-Zn-Mg-Cu series alloys are widely used as aeronautical materials mainly because of their high specific strengths and fracture toughness [1,2,3]. However, this series of aluminum alloys is highly susceptible to intergranular corrosion (IGC), exfoliation corrosion (EXCO) and stress corrosion crack (SCC) [4,5,6]. To enhance the corrosion resistance, one commonly used method is to acquire the anti-corrosion precipitated microstructure by heat processing, such as the over aging or multi-step aging heat treatments. Through these heat treatments, coarsening precipitates and the grain boundary phases (GBPs) with a discontinuous distribution can be obtained, effectively improving the corrosion properties based on either anodic oxidation or hydrogen embrittlement theories [7,8,9,10]. Another method is to inhibit the recrystallization process of deformed aluminum, because the low-angle grain boundaries (LAGBs) within non-recrystallized grains are more resistant to corrosion than the high-angle grain boundaries (HAGBs) within recrystallized grains [11,12]. The addition of minor elements such as Sc and Zr can be used for recrystallization inhibition and can finally promote the improvement of corrosion resistance, which has been continuously reported in the recent literature [13,14,15,16].

In 7000 series alloys with the Sc and Zr additions, the primary Al_3_(Sc_1−x_Zr_x_) phase developing during the casting process can promote the heterogeneous nucleation, consequently benefiting grain refinement [17,18]. The secondary Al_3_(Sc_1−x_Zr_x_) (L1_2_ type) precipitate phase, highly coherent to matrix, usually develops during homogenization [19,20]. It can pin the dislocations and prevent the migration of the grain or subgrain boundary, thereby effectively inhibiting recrystallization [17,21,22,23]. The precipitate phases at the non-recrystallized grain boundary usually have a low nucleation rate during the aging process, due to the lower energy within LAGBs. As a result, the grain boundary phases are more likely to precipitate with a discontinuous distribution at the non-recrystallized grain boundaries [14,18,24]. This structure with discontinuous GBPs has been repeatedly confirmed to be in favor of reducing the intergranular corrosion (IGC) or stress corrosion crack susceptibilities. Based on this, some Al-Zn-Mg alloys acquired markedly enhanced corrosion resistance by the composite addition of Sc and Zr [13,24].

The previous works mainly focused on the overall performance of SCC of the Al alloy materials with some shapes such as the sheet or the bar [13,14,16,18], or focused on the IGC performance of the material surface layer [13,15]. However, the researchers paid much less attention to the effect of gradient microstructures within a material from the surface layer to the central layer on the corrosion behavior. It is well known that the strain inhomogeneity during hot or cold works can finally induce different recrystallization degrees within different thickness layers in the deformed aluminum alloy materials. Taking the extrusion material, for instance, the surface layer under the severer shear deformation will more easily recrystallize, thus leading to a higher recrystallization degree [25,26]. In this work, Zr and Sc were separately or compositely added into the Al-Zn-Mg-Cu alloy with high zinc content s, resulting in the development of gradient structures with various recrystallization degrees in the extruded alloys during the solution treatment process. The research focused on the recrystallization behavior of Al-Zn-Mg-Cu alloys impacted by the addition of Sc and Zr and the influence of gradient microstructures on intergranular corrosion behavior. The electrochemistry parameters of different alloys in different thickness layers were measured to find the correlation between the recrystallized microstructure and corrosion susceptibility. We paid more attention to the IGC behavior of the alloys under the naturally aged condition. In this case, the quenched precipitated GBPs affected by the grain boundary character would play a dominant role in IGC while no obvious precipitates formed in the grain interior, whose influence on IGC could be excluded. Moreover, the effect of primary Al_3_(Sc_1−x_Zr_x_) phases on the localized corrosion behavior was explored, which was rarely researched in previous works.

## 2. Materials and Methods

**Figure 1:**
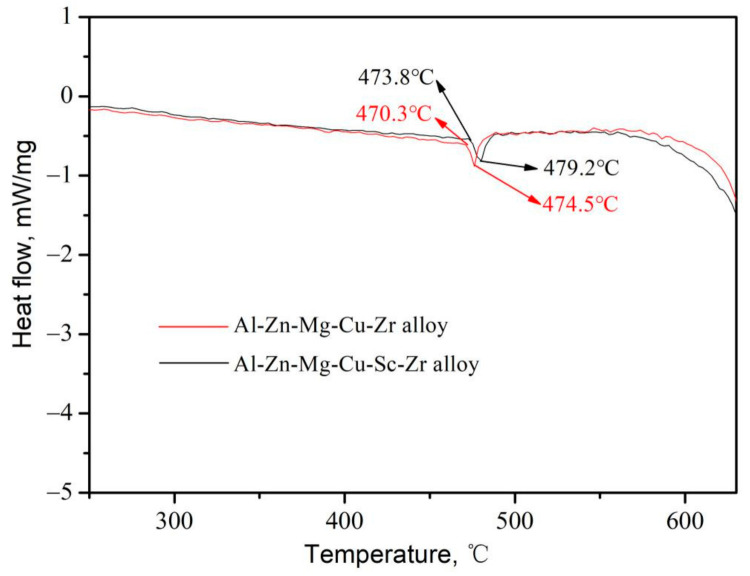
DSC curves of Al-Zn-Mg-Cu alloys in the as-cast condition.

The unique Zr and the composited Sc and Zr were added into a high zinc content Al-Zn-Mg-Cu alloy, respectively. The acquired alloys’ chemical compositions are represented in Table 1. Pure Al was melted at 770 °C, and then Al-50Cu, Al-10Zr, and Al-2Sc were added into the melt. The pure Zn and Mg were added at the last to avoid excess volatilization or burning loss. The melt was stirred well and degassed, and then was held for 15 min. Next, the heating power was cut off and the melt was cast into a cylindrical cast iron mold when the melt temperature decreased to 730 °C, cooling in air with the rate around 10 °C/s. The alloys, in as-cast condition, had a cylindrical shape whose diameter was 100 mm. Figure 1 shows the differential scanning calorimetry (DSC) curves of as-cast alloys. There is an endothermic peak in Al-Zn-Mg-Cu-Zr alloy and Al-Zn-Mg-Cu-Sc-Zr alloy around 474.5 °C and 479.2 °C, respectively. It is known that the endothermic peaks represent the melting of non-equilibrium phases. To avoid the alloy overburning, the highest homogenization temperatures should be inferior to the melting temperature of non-equilibrium phases, namely the starting temperature of the endothermic peak. In this work, the homogenization temperature for Al-Zn-Mg-Cu-Zr alloy and Al-Zn-Mg-Cu-Sc-Zr alloy should be below 470.3 °C and 473.8 °C, respectively. In this experiment, the two alloys were subjected to the double-stage homogenization treatments of (380 °C/6 h + 465 °C/18 h) and (380 °C/6 h + 470 °C /18 h), respectively. The first stage of homogenization at the relatively low temperature was to promote the dispersive precipitation of Al_3_Zr or Al_3_(Sc_1−x_Zr_x_) second phases [27,28]. And then, the homogenized alloys warmed at 430 °C were extruded to some thin plates with a cross-section of 60 mm × 6 mm at the extrusion speed of 1 mm/s, while the extrusion cylinder temperature was 410 °C. Cooling the extruded plates in the water, these extrusions underwent the solution treatments at 470 °C for 30–600 min, then were water quenched. Finally, the extrusions treated at 470 °C for four hours were selected to be aged naturally (T4 conditioned) for ten days at least.

The samples for the microstructure characterization and IGC corrosion experiments were taken from the plates along the central axis of the longitudinal direction. The Vickers hardness tests for the samples that underwent the different solid solution treatments were carried out on a Zwick/Roell machine (Indentec hardness testing machine limited, West Midlands, UK) and the hardness result of each piece was obtained by calculating the average of nine measured values. The samples for optical observation were prepared by mechanical polishing and etching with Keller’s reagent for 10–20 s. The samples for the scanning electron microscope (SEM) observations were also prepared by mechanical polishing and the secondary phase particle distributions in extruded alloys and the corrosion depths and paths in naturally aged alloys were identified through a Zeiss GeminiSEM 300 field emission scanning electron microscope (SEM) (Carl Zeiss AG, Oberkochen, Germany) with energy dispersive spectroscopy (EDS). The microstructural observations of the naturally aged samples and corresponding selected area electron diffraction (SAED) analysis in various conditions were performed by Jeol Jem2100F field emission transmission electron microscopy (TEM) (JEOL, Tokyo, Japan). EBSD experiment samples were prepared by mechanical polish and subsequent ion beam polishing. All EBSD experiments were also carried out on Zeiss GeminiSEM 300 field emission SEM.

IGC tests were carried out following GB/T7998-2005 [29]. IGC depths and paths were measured or characterized by SEM observation. Electrochemical measurements were conducted on a Gamry electrochemical tester with a three electrode system. This electrode system contains the reference electrode (saturated calomel electrode (SCE)), the counter electrode (platinum), and the working electrode (tested samples with a size of 10 mm × 10 mm). Electrochemical impedance spectroscopy (EIS) was measured at the open circuit potential in the frequency range from 0.1 Hz to 100 kHz under the AC signal with 5 mV amplitude. The potentiodynamic polarization curves were measured in the potential range from −1.1V_SCE_ to −0.2V_SCE_ with the scanning rate of 0.5 mV/s. All the electrochemical measurements were carried out in a testing solution with 3.5 wt.% NaCl.

## 3. Results

### 3.1. Microstructure

**Figure 2:**
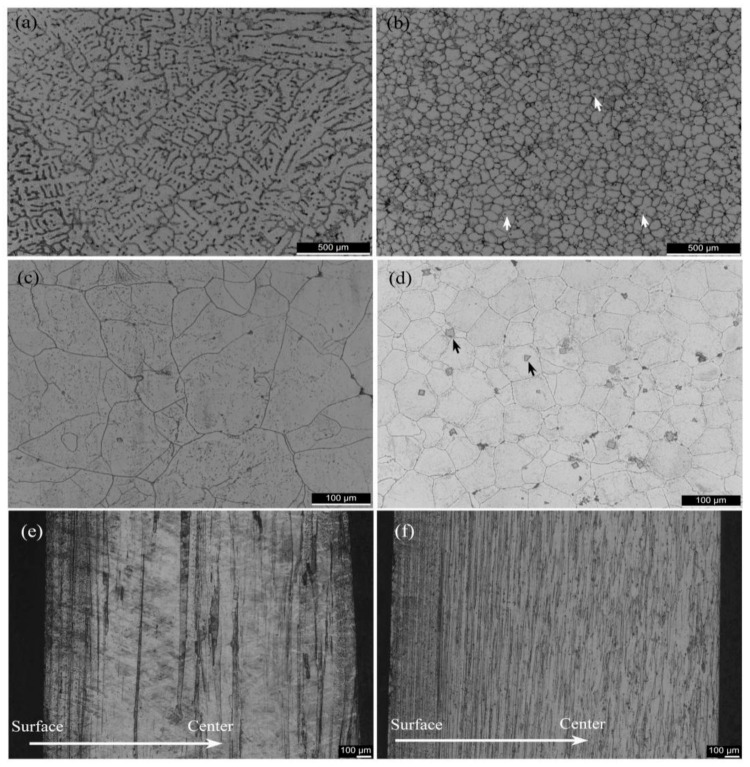
Optical micrographs illustrating the grain morphology of Al-Zn-Mg-Cu alloys. (**a**,**c**,**e**) Al-Zn-Mg-Cu-Zr alloy in the as-cast, homogenization, and hot-extrusion condition, respectively, (**b**,**d**,**f**) Al-Zn-Mg-Cu-Sc-Zr alloy in the as-cast, homogenization, and hot-extrusion condition, respectively.

The optical micrographs of Al-Zn-Mg-Cu-Zr and Al-Zn-Mg-Cu-Sc-Zr alloys in as-cast, homogenization, and extruding conditions are present in Figure 2. Apparently, Al-Zn-Mg-Cu-Zr alloy in the as-cast condition presented a dendritic structure while Al-Zn-Mg-Cu-Sc-Zr alloy mainly consisted of numerous small-size equiaxial grains, as shown in Figure 2a,b. A fair number of the primary Al_3_(Sc_1−x_Zr_x_) phases (arrowed) were able to promote remarkable grain refinement, as seen in Figure 2b. By means of homogenization, the dendritic structure disappeared in Al-Zn-Mg-Cu-Zr alloy. In contrast, the grain morphology in Al-Zn-Mg-Cu-Sc-Zr alloy showed no obvious change during homogenization. Besides, the non-equilibrium solidification brings about the generation of non-equilibrium phases and the non-equilibrium phases tend to be more concentrated on the grain boundaries. After the homogenization, the grain boundary became thinner and smoother, which suggests that the majority of non-equilibrium phases had dissolved, as seen in Figure 2c,d. The primary Al_3_(Sc_1−x_Zr_x_) particles (arrowed) remained stable in this process. Figure 2e,f shows the microstructures in the longitudinal section of the two alloys in the extruded condition. Both alloys consisted of deformed grain fibers, but Al-Zn-Mg-Cu-Sc-Zr alloy showed thinner grain sizes. It can be found that in both alloys the surface layer undergoing more serious deformation showed much thinner grain fibers in comparison with the central layer, indicating a deformation gradient existing from the plate surface to the inner plate.

**Figure 3:**
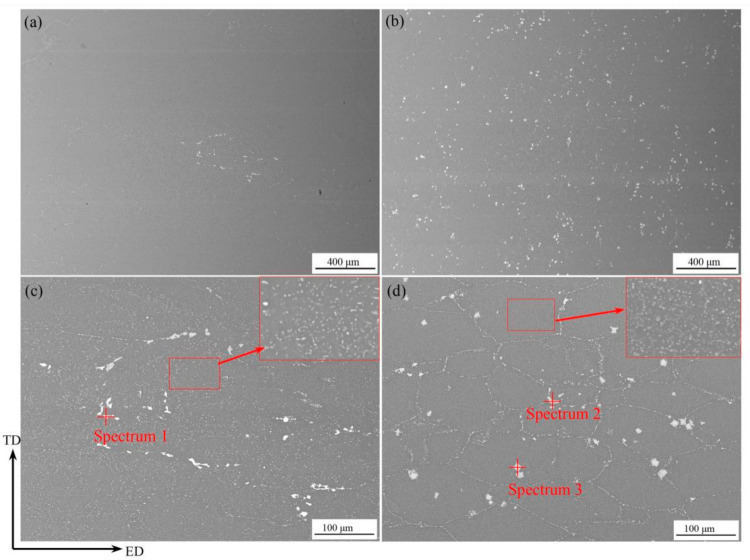
BSE images of secondary phases of Al-Zn-Mg-Cu-Zr alloy (**a**,**c**) and Al-Zn-Mg-Cu-Sc-Zr alloy (**b**,**d**) in the hot-extrusion condition.

It is known that the secondary phase or intermetallic compounds can exert dramatic influence on recrystallization, so the distribution of secondary phase Al-Zn-Mg-Cu alloys in the hot-extrusion condition were characterized by SEM, as seen in Figure 3. The observation plane is parallel to the ED-TD plane. Table 2 represents EDS analysis results of secondary phases. From Figure 3a,b, it can be found that more secondary particles are distributed in Al-Zn-Mg-Cu-Sc-Zr alloy. The backscattered electron (BSE) images in the large magnification show that in both alloys the large intermetallics were mainly located at the grain boundaries or the intersection between grain boundaries while the much finer dispersive precipitations were distributed in the grain interior, as shown in Figure 3c,d. For Al-Zn-Mg-Cu-Zr alloy, according to the result of EDS analysis, the large intermetallic (spectrum 1) was suspected to be the Mg(Zn, Al, Cu)_2_ particle, one of the residual non-equilibrium phases that were widely mentioned in the previous investigation about the high Zn content Al-Zn-Mg-Cu alloys [30,31,32]. For Al-Zn-Mg-Cu-Sc-Zr alloy, the residual non-equilibrium phase (spectrum 2) should be the Mg(Zn, Al, Cu)_2_ particle with a different atom ratio, but the intermetallic particles (spectrum 3) with the square shape remained both at grain boundary and in the grain interior, which should be the primary Al_3_(Sc_1−x_Zr_x_) phase with high stability [33,34]. Besides, the fine secondary phase in the grain interior of both alloys, which could not be detected accurately by EDS analysis, was probably the precipitated MgZn_2_ (η) phase at high temperature during the hot extrusion processes [30,32].

**Figure 4:**
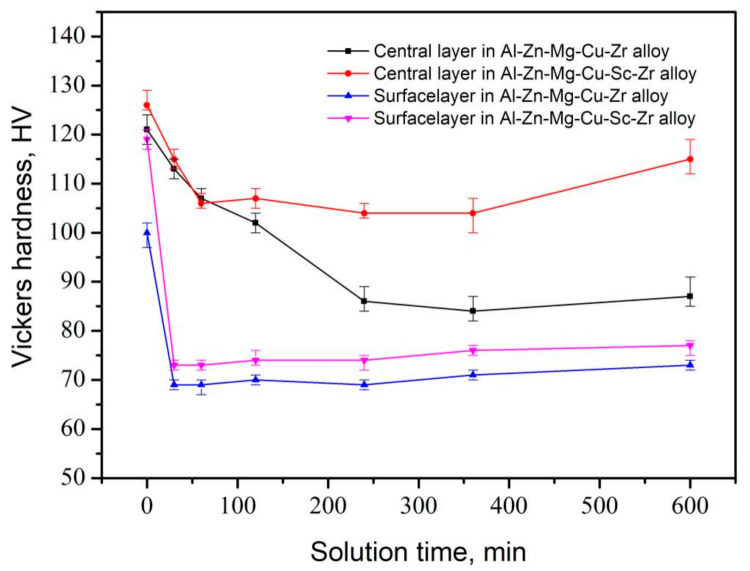
Vickers hardness variation curves of Al-Zn-Mg-Cu alloys in the central and surface layers as the solution time is prolonged at 470 °C.

Figure 4 represents Vickers hardness test results for the samples that underwent the different solid solution treatments. It can be clearly seen that there were different variation tendencies of hardness between the surface layer and the central layer of the alloy extrusions. The Vickers hardness of the surface layer decreased more sharply with the solution time increasing than the central layer. For example, as the solution time increased from 0 min to 240 min, for Al-Zn-Mg-Cu-Zr alloy, the central hardness decreased from 121 HV to 86 HV, while the surface hardness decreased from 126 HV to 104 HV. For Al-Zn-Mg-Cu-Sc-Zr alloy, the surface hardness also decreased greatly from 119 HV to 74 HV, but a smaller drop was observed in the hardness curves of the central layer. The distinct decreases of surface hardness in both alloys indicated the obvious activated recrystallization process. The large decrease of the central layer in Al-Zn-Mg-Cu-Zr alloy also suggested that the obvious recrystallization should occur. However, Al-Zn-Mg-Cu-Sc-Zr alloy showed a smaller hardness decrease in the central layer. This means that the recrystallization degree was low or even that the recrystallization did not happen, but the recovery occurred instead [35]. Besides, the initial hardness for Al-Zn-Mg-Cu-Zr alloy in the surface was much lower than that in the center, suggesting that a dynamic softening process such as dynamic recovery or dynamic recrystallization probably occurred during the hot extrusion [36].

**Figure 5:**
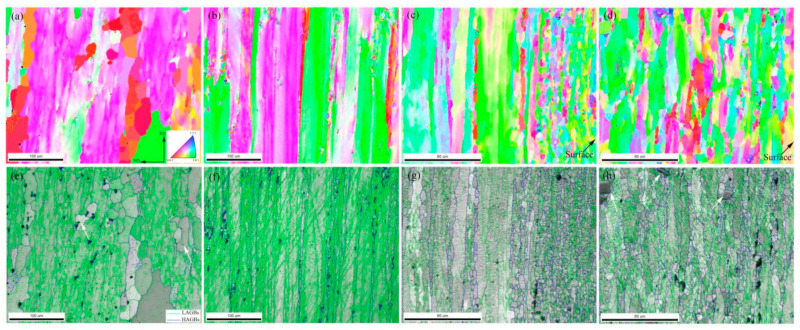
Grain orientation and grain boundary distribution maps of Al-Zn-Mg-Cu alloys after the solution treatment. (**a**,**e**) Al-Zn-Mg-Cu-Zr alloy in the central layer, (**b**,**f**) Al-Zn-Mg-Cu-Sc-Zr in the central layer, (**c**,**g**) Al-Zn-Mg-Cu-Zr alloy in the surface layer, (**d**,**h**) Al-Zn-Mg-Cu-Sc-Zr alloy in the surface layer.

**Figure 6:**
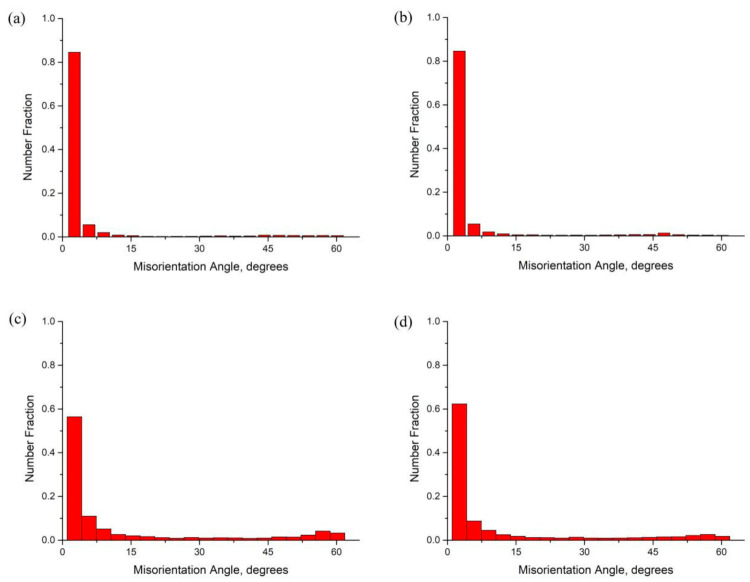
Misorientation angle distributions of Al-Zn-Mg-Cu-Zr alloy in the central layer (**a**), Al-Zn-Mg-Cu-Sc-Zr alloy in the central layer (**b**), Al-Zn-Mg-Cu-Zr alloy in the surface layer (**c**), and Al-Zn-Mg-Cu-Sc-Zr alloy in the surface layer (**d**).

Figure 5 shows EBSD maps of the central and surface layers in each extruded alloy undergoing the 470 °C/4 h solution and natural aging treatments. It is known that the boundaries of recrystallized grains are mainly the high-angle grain boundaries (HAGBs), and the grains usually represent a near equiaxed shape. In contrast, the boundaries of non-recrystallized deforming grains are mainly the low-angle (sub)grain boundaries (LAGBs), and the grains represent an elongated shape. Therefore, the recrystallization degree can be regarded as being positively correlated with the proportion of HAGBs. Besides, the dislocation or substructure density in the recrystallized grains interior is relatively lower than that in the non-recrystallized grains. So it is easy to differentiate the recrystallized and non-recrystallized grains from the grain boundary angle, grain morphology, and intragranular dislocation or substructure density. In this work, the proportion of HAGBs was used to evaluate the height of recrystallization degree. In the grain boundary distribution maps shown in Figure 5e,f, the blue lines represent HAGBs while the green lines represent LAGBs. According to Figure 5a,b,e,f, it can be found that there was an obvious difference in the recrystallization degree between Al-Zn-Mg-Cu-Zr alloy and Al-Zn-Mg-Cu-Sc-Zr alloy in the central layer. Al-Zn-Mg-Cu-Zr alloy represented an obvious recrystallization in the central layer, due to the newborn grains developing with many HAGBs, as shown in Figure 5e. Noticeably, there were few substructures in the interior of the grains as arrowed in Figure 5e, indicating that this grain with HAGBs should be a recrystallized grain. In contrast, as shown in Figure 5f, in the central layer of the Al-Zn-Mg-Cu-Sc-Zr alloy extruded plate, the original fibrous deformed grains mostly remained and only some small recrystallized grains developed during the solution process, representing a low degree of recrystallization. Since the surface layer underwent a severer deformation, both of the alloys showed a greater recrystallization degree in the surface layer, as shown in Figure 5c,d,g,h. For Al-Zn-Mg-Cu-Zr alloy, a conspicuous recrystallized grain band with approximate 60 μm width, consisting of numerous refined grains, can be observed in the area close to the surface. In contrast, a partially recrystallized microstructure can be seen in the deeper layer, including the non-recrystallized deformed grains. There was no distinct transition boundary between the deformed and recrystallized grains in Al-Zn-Mg-Cu-Sc-Zr alloy. However, a declined gradient of recrystallization degree still existed from the surface to the inner layer. Figure 6 represents the misorientation angle distributions in the different thickness layers of the two extruded alloys. The boundaries with the misorientation angles superior to 15° can be classified into HAGBs. The results show that the HAGB proportions of Al-Zn-Mg-Cu-Zr alloy and Al-Zn-Mg-Cu-Sc-Zr alloy in the surface layer were 7.21% and 7.14%, respectively. It was known that a large part of the HAGBs in the Al-Zn-Mg-Cu-Sc-Zr alloy was actually the original boundaries of deformed grains (Figure 5f). Therefore, even though the HAGB proportion values of the two alloys were very close to each other, it can be supposed that the recrystallization degree in Al-Zn-Mg-Cu-Sc-Zr alloy should be much smaller than that in Al-Zn-Mg-Cu-Zr alloy. In the central layer, the HAGB proportions of Al-Zn-Mg-Cu-Zr alloy and Al-Zn-Mg-Cu-Sc-Zr alloy were 23.92% and 21.01%, respectively. This indicates that the recrystallization degrees of the selected areas in the two alloys were close to each other. However, it should be noted that the recrystallized grains were mainly concentrated in the region close to the surface in Al-Zn-Mg-Cu-Zr alloy. Apparently, in this region, the higher degree of recrystallization occurred in Al-Zn-Mg-Cu-Zr alloy. Besides, it can be observed that the recrystallized grains developed into the deeper layer of Al-Zn-Mg-Cu-Sc-Zr alloy. This should most probably be attributed to the coarse primary Al_3_(Sc_1−x_Zr_x_) phases which have a promoting effect on the recrystallization due to the particle-stimulated nucleation (PSN) [37,38,39] mechanism. As indicated in Figure 5h, the small recrystallized grains developed close to the coarse primary Al_3_(Sc_1−x_Zr_x_) phases (arrowed).

**Figure 7:**
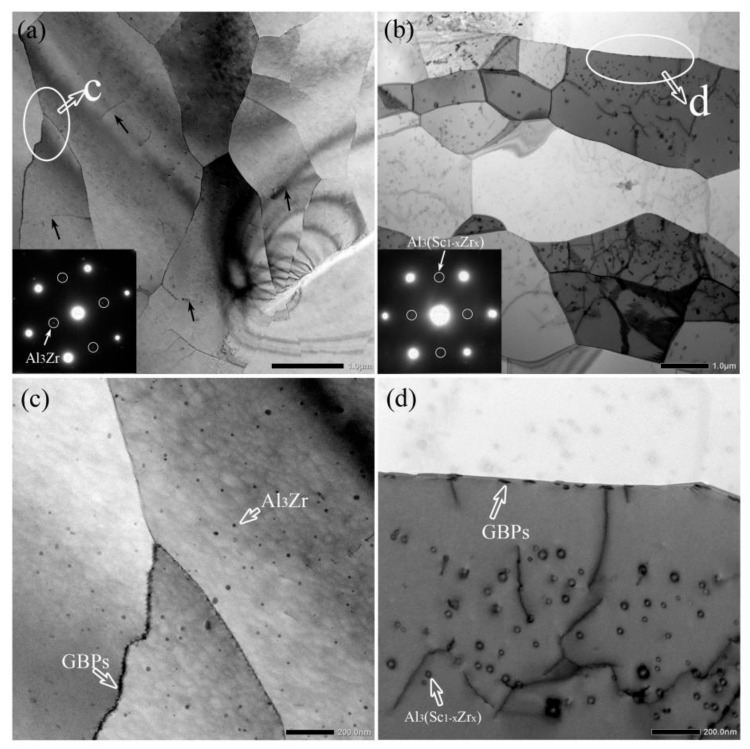
TEM bright field micrographs and corresponding SAED patterns of naturally aged Al-Zn-Mg-Cu alloys in the intermediate layer. (**a**,**c**) Al-Zn-Mg-Cu-Zr alloy, (**b**,**d**) Al-Zn-Mg-Cu-Sc-Zr alloy.

Figure 7 shows the TEM microstructure images of intermediate (around 1/4 thickness) layers of the naturally aged extruding alloys. Figure 7c,d corresponds to the ellipse regions of Figure 7a,b. The SAED patterns reflect the phase information of the grains with a dark contrast in Figure 7a,b. The SAED patterns, with a distinct superlattice characteristic, indicate that numerous nanometer-scale coherent Al_3_Zr and Al_3_(Sc_1__−__x_Zr_x_) secondary phases should exist in Al-Zn-Mg-Cu-Zr and Al-Zn-Mg-Cu-Sc-Zr alloy, respectively. It can be found that the Al_3_(Sc_1__−__x_Zr_x_) phases showed a similar amount but slightly larger sizes in Al-Zn-Mg-Cu-Sc-Zr alloy than the Al_3_Zr phases in Al-Zn-Mg-Cu-Zr alloy, as shown in Figure 7c,d. This should be the result of the addition of Sc element in Al-Zn-Mg-Cu-Sc-Zr alloy. Moreover, it can be observed that few dislocations remained in the grain interior and the subgrains tended to be merged into a large grain, in which the original subgrain boundary (arrowed) tended to disappear, as shown in Figure 7a. This indicates that obvious recrystallization behavior should occur in Al-Zn-Mg-Cu-Zr alloy during the solution process. In contrast, the naturally aged Al-Zn-Mg-Cu-Sc-Zr alloy remained the microstructure with numerous subgrains in which an appreciable number of dislocations were retained, still pinned by the secondary Al_3_(Sc_1__−__x_Zr_x_) particles, as shown in Figure 7b. Besides, the grain boundary precipitates (GBPs) can be observed in both alloys. It is apparent that GBPs in Al-Zn-Mg-Cu-Zr alloy tended to form with a continuous distribution on the recrystallized grain boundary as shown in Figure 7c. In contrast, relatively fewer GBPs formed on the grain boundary, as shown in Figure 7b, and most of them were located at the subgrain boundaries with a discontinuous distribution, as shown in Figure 7d. This suggests that GBPs in Al-Zn-Mg-Cu-Zr alloy should possess a lower nucleation rate during the quenching process.

### 3.2. IGC Behavior

**Figure 8:**
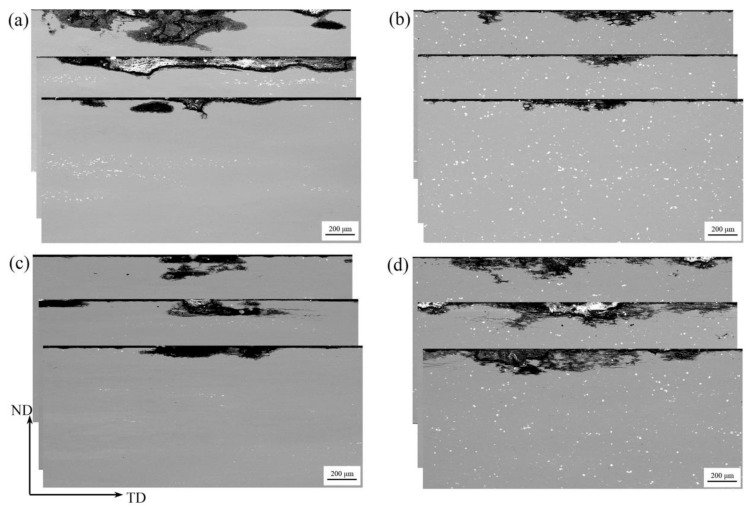
BSE images of representative intergranular corrosion area by SEM observation. (**a**) Al-Zn-Mg-Cu-Zr alloy in the central layer, (**b**) Al-Zn-Mg-Cu-Sc-Zr alloy in the central layer, (**c**) Al-Zn-Mg-Cu-Zr alloy in the surface layer, (**d**) Al-Zn-Mg-Cu-Sc-Zr alloy in the surface layer.

The IGC test results of the extruded alloys after the natural aging are present in Figure 8. The representative intergranular corrosion sections were observed by SEM. The observation plane is parallel to the TD-ND plane. Table 3 represents the variation ranges of the corrosion depths and corrosion widths measured at the three corrosion locations with relatively large depths. Obviously, Al-Zn-Mg-Cu-Sc-Zr alloy in the central layer represented the lowest intergranular corrosion susceptibility, due to the smallest corrosion depth and width. For Al-Zn-Mg-Cu-Zr alloy, the surface layer showed not much difference in IGC susceptibility as compared to its central layer, according to Figure 8a,c. In contrast, for Al-Zn-Mg-Cu-Sc-Zr alloy, the surface layer showed higher IGC susceptibility than the central layer, according to Figure 8b,d. In addition, this IGC susceptibility of the surface layer in Al-Zn-Mg-Cu-Sc-Zr alloy even seems the highest among all layers of various alloys.

**Figure 9:**
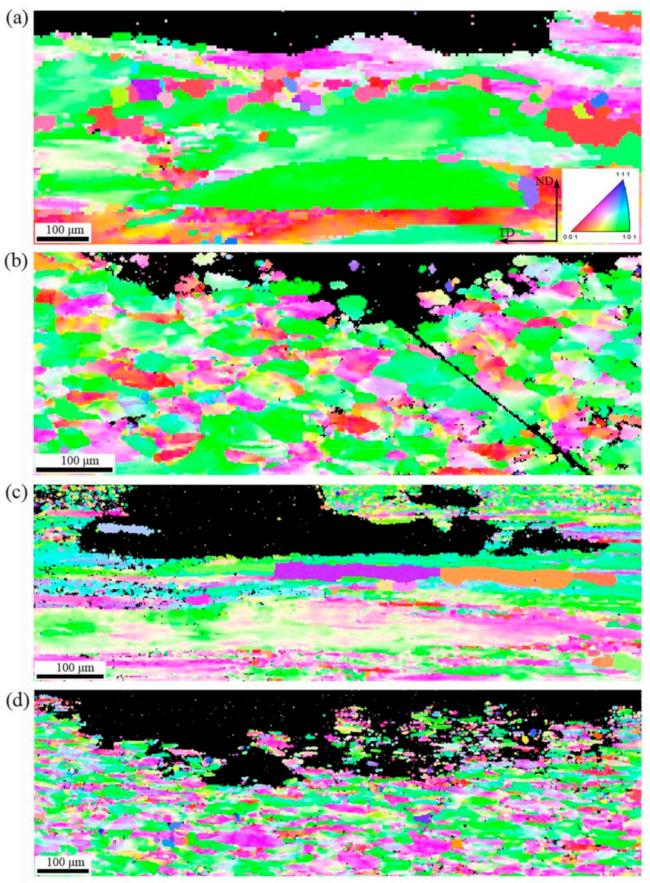
Grain orientation maps of IGC samples in the transverse section. (**a**) Al-Zn-Mg-Cu-Zr alloy in the central layer, (**b**) Al-Zn-Mg-Cu-Sc-Zr in the central layer, (**c**) Al-Zn-Mg-Cu-Zr alloy in the surface layer, (**d**) Al-Zn-Mg-Cu-Sc-Zr alloy in the surface layer.

Figure 9 shows the EBSD maps in the cross-sections of the samples undergoing the IGC test, containing the representative intergranular corrosion area. For Al-Zn-Mg-Cu-Zr alloy, the corrosion interface at the maximum depth almost developed along the grain boundaries of the elongated deformed grains, as shown in Figure 9a,c. In particular, in the surface layer, the corrosion mainly occurred in the area dominantly consisting of the refined recrystallized grains. Figure 9c shows that the corrosion depth did not continue increasing when the corrosion reached the grain boundary of non-recrystallized grains, but instead, the corrosion would continue at the grain boundaries along the transverse direction. For Al-Zn-Mg-Cu-Sc-Zr alloy, the corrosion with a more distinguishing IGC characteristic was observed in different depth layers, as shown in Figure 9b,d. The uncorroded grain interior remained even though the corrosion had developed into a larger depth area. Besides, more small recrystallized grains in the surface layer either for Al-Zn-Mg-Cu-Zr or Al-Zn-Mg-Cu-Sc-Zr alloy suggested that IGC in the surface area should own a longer corrosion path.

### 3.3. Electrochemical Characteristics

**Figure 10:**
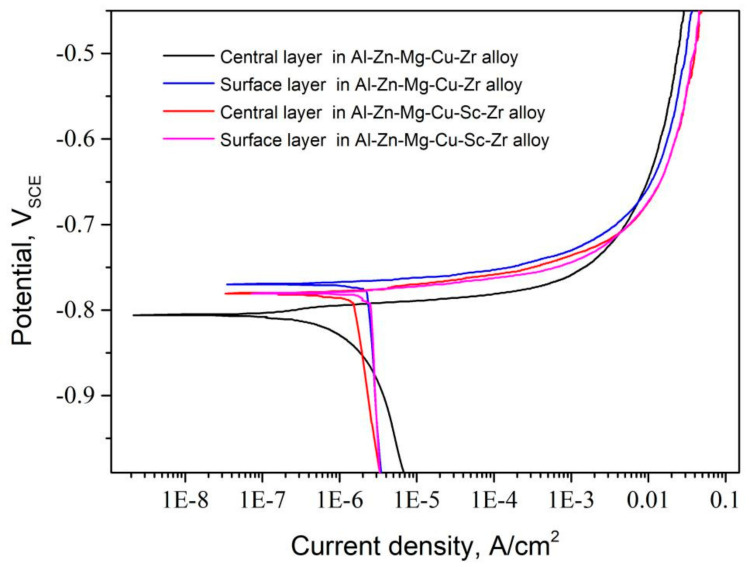
Potentiodynamic polarization curves of the central and surface layer samples of Al-Zn-Mg-Cu alloys in the natural aging condition.

Figure 10 shows the polarization curves of the extruded alloys in the different thickness layers after naturally aging. Table 4 represents the evaluated results of the electrochemical corrosion parameters including the corrosion potential (E_corr_) and the corrosion current density (I_corr_). Polarization curves of various samples showed a similar development trend with the potential increasing, but there were still apparent differences in detail. The sample for the central layer of Al-Zn-Mg-Cu-Zr alloy showed the most negative E_corr_, while the sample for the surface layer of the same alloy represented the highest E_corr_. For Al-Zn-Mg-Cu-Sc-Zr alloy, the corrosion potentials of the surface and central layer samples were close to each other. It has been reported that the corrosion current density I_corr_ is linearly proportional to the electrochemical corrosion speed by Faraday’s law [13,40]. Thereby the lowest I_corr_ in the central layer sample of Al-Zn-Mg-Cu-Sc-Zr alloy suggested the slowest electrochemical corrosion occurring in this layer. Interestingly, the fastest electrochemical corrosion also happened in this alloy but in the surface layer. In general, the surface layer samples at all times show the faster corrosion speed, but Al-Zn-Mg-Cu-Zr alloy with a relatively higher recrystallization degree in the surface layer strangely represented a lower corrosion speed as compared to Al-Zn-Mg-Cu-Sc-Zr alloy.

**Figure 11:**
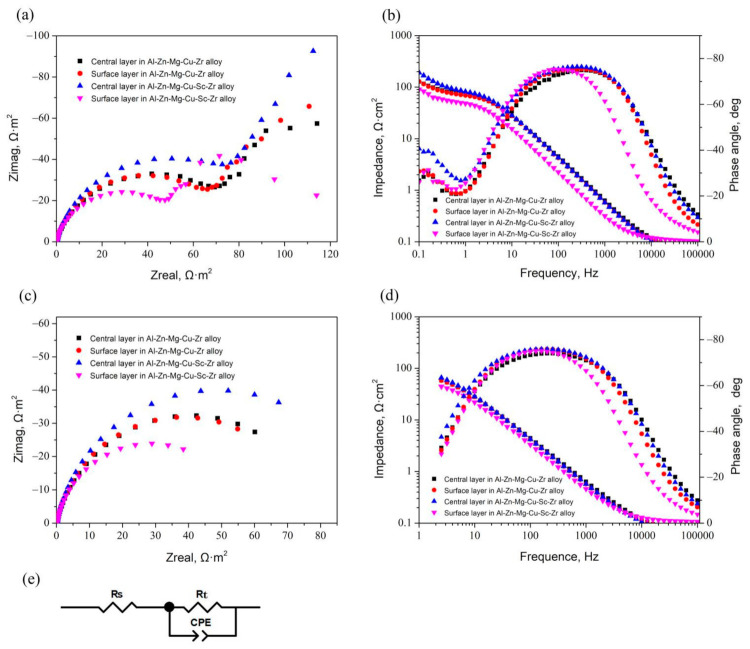
EIS spectra of the central and surface layer samples of Al-Zn-Mg-Cu alloys in the natural aging condition. (**a**) Measured Nyquist plots, (**b**) measured Bode plots, (**c**) fitted Nyquist plots in the high-frequency range, (**d**) fitted Bode plots in the high-frequency range, (**e**) the equivalent circuit diagram.

The measured and fitted Nyquist and Bode plots of the two extruded alloys after naturally aging are present in Figure 11. From the measured results, as shown in Figure 11a,b, it can be found that all the plots can be divided into two parts, the high-frequency part and the low-frequency part. In the Nyquist plot, the capacitive loop in the relatively smaller Z_real_ range corresponded to the charge-transfer-controlled electrode process in the high-frequency range, while the remainder containing a line segment with near 45° slope in the larger Z_real_ range corresponded to the diffusion-controlled electrode process in the low-frequency range. Similarly, in the Bode plot, an additional time constant was represented in the low-frequency range due to the occurrence of the diffusion-controlling electrode process. The radius of the loop in the high-frequency range was supposed to be related to the charge-transfer resistance (R_t_). In particular, in the fundamental R-C circuit, the loop radius value is actually equal to R_t_. Besides, the Bode plots can also reflect the variation of the charge-transfer resistance, because the variation of the maximum phase angle in the height or width always keeps pace with the variation of the charge-transfer resistance. Considering that R_t_ is inversely proportional to I_corr_ [13,14,41,42], which can reflect the corrosion resistance, the charge-transfer-controlled electrode process in the high-frequency range was the main focus in this investigation. The fitting Nyquist and Bode plots based on the fundamental R-C circuit are represented in Figure 11c,d. This R-C equivalent circuit consists of three elements, including solution resistance (R_s_), charge transfer resistance (R_t_), and constant phase element (CPE) [27,42], as shown in Figure 11e. This could fit the measured results well in the high-frequency range. Based on this circuit, the fitting results of EIS parameters are represented in Table 5. It can be found that the R_s_ values of all the samples were far lower than their R_t_ values, indicating that R_s_ could be neglected. The CPE-n values were all in the interval of 0.5~1, suggesting that all the constant phase elements were actually capacitive elements, such as the equivalence element of the double-layer capacitance. All CPE-T values were at a lower level, meaning that the double-layer capacitances were small, but the CPE-T value of the surface layer sample of Al-Zn-Mg-Cu-Sc-Zr alloy was much smaller than the other samples. Since R_t_ is proportional to the corrosion resistance, the central layer sample of Al-Zn-Mg-Cu-Sc-Zr alloy possessing the largest R_t_ value indicates that the central layer of this alloy should have the highest corrosion resistance. Moreover, it can be found that the fitted R_t_ results in the EIS analysis were consistent with the results of corrosion’s current density in the polarization curve analysis.

## 4. Discussion

### 4.1. Microstructures and Recrystallization Behavior

The effect of Sc and Zr additions on the microstructure of Al-Zn-Mg-Cu alloy is mainly embodied in two aspects, including grain refinement and recrystallization inhibition. In this research, the addition of Sc brought about a more obvious grain refinement effect on as-cast Al-Zn-Mg-Cu-Sc-Zr alloy, as compared to no scandium addition, as shown in Figure 2a,b. Due to the relatively high content of Sc element, a considerable number of primary Al_3_(Sc_1−x_Zr_x_) phases were generated during the solidification process, as shown in Figure 2b. These primary phases were able to promote heterogeneous nucleation and consequently brought about grain refinement. The original grain size would directly affect the grain morphology of the subsequent hot extruded alloy. Due to the grain refinement of Al-Zn-Mg-Cu-Sc-Zr alloy in as-cast condition, the fiber-like deformed grains of this alloy in extruded condition were shown to be thinner, as shown in Figure 2e,f.

The addition of Sc and Zr on the recrystallization inhibition could be actualized by the precipitation of Al_3_Zr or Al_3_(Sc_1−x_Zr_x_) phases which have high coherency to the aluminum matrix. It has been reported that the second Al_3_Zr or Al_3_(Sc_1−x_Zr_x_) particles can precipitate more dispersively by the double stage homogenization treatment [22,27,43]. These coherent particles could effectively pin the dislocations and prevent the grain boundary migration, and finally the recrystallization would be inhibited. However, the inhibiting effect of Al_3_Zr and Al_3_(Sc_1−x_Zr_x_) particles on recrystallization showed an obvious difference in this work. From Figure 4, it can be seen that a significant decrease of Vickers hardness was represented in the central layer of Al-Zn-Mg-Cu-Zr alloy, but a small decrease was shown in the central layer of Al-Zn-Mg-Cu-Sc-Zr alloy, indicating that the recrystallization in the former alloy should more easily be activated during the solution process. Then the EBSD analysis results shown in Figure 5a,b,e,f confirm that an obvious recrystallization occurred in Al-Zn-Mg-Cu-Zr alloy, while the recrystallization degree in Al-Zn-Mg-Cu-Sc-Zr alloy was relatively low. In the surface layer of the extrusions, large drops in the Vickers hardness were represented in both alloys, suggesting obvious recrystallization should occur in the two alloys. EBSD analysis results shown in Figure 5c,d,g,h confirm that the surface layer microstructures in both alloys were partially recrystallized. In addition, a relatively higher degree of recrystallization occurred in the area close to the extrusion surface in Al-Zn-Mg-Cu-Zr alloy. Based on the above, it can be summarized that the recrystallization process was more easily activated in Al-Zn-Mg-Cu-Zr alloy. The reason should be attributed to the difference in ability of Al_3_Zr and Al_3_(Sc_1−x_Zr_x_) in pinning the dislocation or the quantity difference of these coherent particles. As shown in Figure 7, the quantities of Al_3_Zr and Al_3_(Sc_1−x_Zr_x_) particles were close to each other, and the Al_3_(Sc_1−x_Zr_x_) particle even showed a larger size than the Al_3_Zr particles. More importantly, the subgrain boundary had been divorced from the pinning of Al_3_Zr, and the subgrains tended to merge by the climb of dislocations on the subgrain boundary. In contrast, the dislocation pinning of Al_3_(Sc_1−x_Zr_x_) remained in Al-Zn-Mg-Cu-Sc-Zr alloy. This suggests that Al_3_(Sc_1−x_Zr_x_) particles should have a stronger ability in inhibiting the recrystallization. It has been reported that the coherent particles forming during the homogenization process will lose their high coherency in the subsequent hot working process [44,45]. Therefore, this means that the Al_3_(Sc_1−x_Zr_x_) particles should have higher stability and smaller coherency loss during the hot extrusion or the solution processes.

Except for the nanoscale coherent particles, the other intermetallic particles with micron sizes were also observed in extruded alloys as shown in Figure 3. It has been reported that the micron-sized particles can stimulate the recrystallization occurring near these particles, because the large strain around the particles promotes a large driving force of recrystallization and offers more recrystallization nucleation sites. This recrystallization mechanism is, namely, particle-stimulated nucleation (PSN) [37,39]. From Figure 3, it can be found that there were more micron-sized particles in Al-Zn-Mg-Cu-Sc-Zr alloy under the extruded condition, especially the primary Al_3_(Sc_1−x_Zr_x_) particles with high stability. Therefore, the recrystallization brought about by PSN should be represented more obviously in Al-Zn-Mg-Cu-Sc-Zr alloy. This can be confirmed from Figure 5d,h, because the fine recrystallized grains indeed developed around the coarse Al_3_(Sc_1−x_Zr_x_) phases. Moreover, due to the PSN effect of Al_3_(Sc_1−x_Zr_x_) particles, the recrystallization can penetrate into a larger depth. It can be found that although this type of recrystallization showed a nonuniformity or discontinuity, it gave rise to the fact that the recrystallization degree of the surface layer in Al-Zn-Mg-Cu-Sc-Zr alloy increased remarkably, even close to that of Al-Zn-Mg-Cu-Zr alloy. As seen in Figure 6c,d, the ratios of HAGBs of Al-Zn-Mg-Cu-Sc-Zr and Al-Zn-Mg-Cu-Sc-Zr alloy in the surface layer were close to each other. This indicates the similar recrystallization degree between the two alloys in the surface layer. However, if the recrystallization induced by PSN was not taken into consideration, the recrystallization degree of Al-Zn-Mg-Cu-Sc-Zr alloy would be much lower than the actual level.

### 4.2. Intergranular Corrosion Behavior

As we know, the grain boundary precipitates (GBPs) have an important influence on intergranular corrosion. Due to their negative potentials to the Al matrix, GBPs act as the anode during the electrochemical corrosion process. The continuous GBP distribution is supposed to promote a corrosion channel along the grain boundary, and finally accelerate the corrosion speed [7,8,14,24]. In this work, because the alloys were treated by natural aging, GBPs mostly precipitated during the quenching process. Although GBPs in the naturally aged alloy were much less than those in the peak-aged or over-aged alloy, there were obvious differences in the precipitation quantity and precipitation distribution between Al-Zn-Mg-Cu-Zr and Al-Zn-Mg-Cu-Sc-Zr alloy, as shown in Figure 7. It is clear that GBPs precipitated with a continuous distribution in Al-Zn-Mg-Cu-Zr alloy, while fewer GBPs with a discontinuous distribution precipitated in Al-Zn-Mg-Cu-Sc-Zr alloy. It is well known that the grain boundary characteristics should have a noticeable impact on the GBPs’ precipitation. More concretely, the recrystallized grain boundaries usually are the high-angle grain boundaries (HAGBs), which can offer more nucleation sites and consequently promote the continuous precipitation of GBPs. In contrast, the non-recrystallized grains offer more low-angle grain boundaries (LAGBs), which can promote the discontinuous precipitation of GBPs. Due to the stronger pinning effect of the second Al_3_(Sc_1−x_Zr_x_) particles on dislocations, the recrystallization in Al-Zn-Mg-Cu-Sc-Zr alloy was inhibited and resulted in lower recrystallization degrees, especially in the central layer, as shown in Figure 5. Therefore, more LAGBs brought about less GBPs precipitation, and these GBPs were more likely to precipitate with the discontinuous distribution, as shown in Figure 7b,d. Consequently, the non-recrystallized grain structure should have a higher corrosion resistance than the recrystallized grain structure. This can be confirmed from IGC and electrochemical measurement results. The central layer of Al-Zn-Mg-Cu-Sc-Zr alloy with the lowest recrystallization degree represented the lowest IGC susceptibility, as shown in Figure 8. The electrochemical measurement results also showed that the central layer sample of Al-Zn-Mg-Cu-Sc-Zr alloy has the largest charge-transfer resistance (R_t_) and the smallest I_corr_, as shown in Figure 10 and Figure 11. Besides, in the surface layer of Al-Zn-Mg-Cu-Zr alloy, the corrosion mainly occurred in the area consisting of the recrystallized grains, as shown in Figure 9c, also suggesting that the non-recrystallized grain structure should possess higher corrosion resistance.

However, the samples of Al-Zn-Mg-Cu-Zr alloy in the central and surface layers represented no significant differences in the corrosion speed or R_t_ value, even though the surface layer was more highly recrystallized. According to the reference [46,47], it probably should be attributed to the grain size difference. As shown in Figure 5a,c, the grains in the surface layers were much smaller than those in the central layer. The smaller grain sizes mean that fewer solute atoms existed in the interior of a single grain, and the total length of grain boundaries increased. This means that fewer solute atoms move to the grain boundary of per unit length for the nucleation and growth of GBPs. As a result, the nucleation and growth rates of GBPs will decrease. Therefore, fewer GBPs precipitate on the per unit length boundary of finer grains. This will promote the discontinuous distribution of GBPs. Finally, the electrochemical corrosion in which the GBPs acted as the anode will be slowed down.

**Figure 12:**
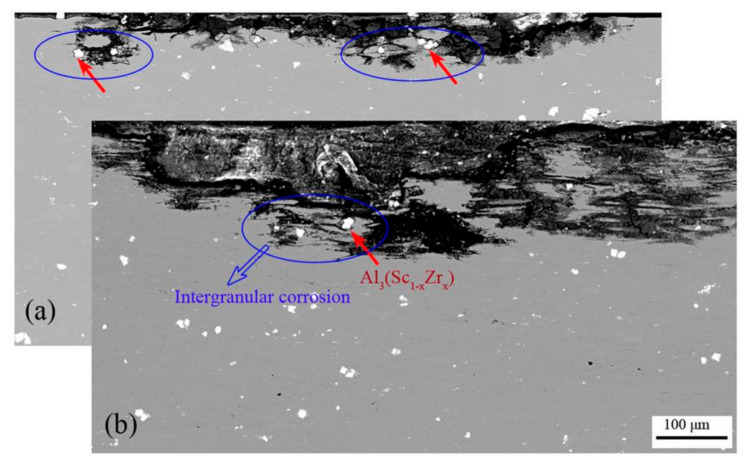
BSE images of the local corrosion regions around the primary Al_3_(Sc_1−x_Zr_x_) phase particles. (**a**) Al-Zn-Mg-Cu-Sc-Zr alloy in the central layer, (**b**) Al-Zn-Mg-Cu-Sc-Zr alloy in the surface layer.

Another dramatic result in this work is that the surface layer sample of Al-Zn-Mg-Cu-Sc-Zr alloy showed the worst corrosion resistance, even though the grain structure close to the surface of the extrusion was less recrystallized as compared to Al-Zn-Mg-Cu-Zr alloy. Figure 12a,b show the images of the local corrosion regions around the primary Al_3_(Sc_1−x_Zr_x_) phase particles, which were taken from Figure 8b,d, respectively. It can be clearly seen that the corrosion occurred around the primary Al_3_(Sc_1−x_Zr_x_) particle with an obvious IGC characteristic. It has been confirmed that Al_3_(Sc_1−x_Zr_x_) phases possess a more positive potential than the aluminum matrix and GBPs [15,34,48]. As a result, the Al_3_(Sc_1−x_Zr_x_) phase particles will act as the cathode while GBPs act as the anode during the electrochemical process. The potential difference between the Al_3_(Sc_1−x_Zr_x_) phase and the GBP phase is more than that between the Al matrix and the GBP phase. Therefore, it is foreseeable that the anode dissolution rate of GBPs should be larger in the electrochemical system of Al_3_(Sc_1−x_Zr_x_) phases (as cathode) and GBPs (as anode) than that in the electrochemical system of Al matrix (as cathode) and GBPs (as anode). In Al-Zn-Mg-Cu-Sc-Zr alloy with a high content of Sc, an appreciable number of non-coherent primary Al_3_(Sc_1−x_Zr_x_) phases could form during the solidification and remain stable in the subsequent thermomechanical processing. Besides, the recrystallization could easily be around the primary Al_3_(Sc_1−x_Zr_x_) particles due to PSN mechanism, as shown in Figure 5d,h. It would have resulted in an accelerated electrochemical process between the primary Al_3_(Sc_1−x_Zr_x_) particles and GBPs on the recrystallized grain boundaries. Based on the above, it is well understood why the surface layer sample of Al-Zn-Mg-Cu-Sc-Zr alloy showed much lower corrosion resistance. However, it can be found that the primary Al_3_(Sc_1−x_Zr_x_) phases also existed in the central layer of Al-Zn-Mg-Cu-Sc-Zr alloy but still showed the lowest IGC susceptibility. It is probably because the low level of recrystallization caused a few GBPs to precipitate with the discontinuous distribution during the quenching process. As a result, this accelerated electrochemical process between the primary Al_3_(Sc_1−x_Zr_x_) particles and GBPs was not significant during the corrosion. Based on the above, it can be summarized that the precipitation of primary Al_3_(Sc_1−x_Zr_x_) phases should be reduced as far as possible to avoid the sharp drop of corrosion resistance. It is well known that the formation of primary Al_3_(Sc_1−x_Zr_x_) phases is based on the peritectic reaction (Al_3_Zr + liquid → α-Al) and the eutectic reaction (liquid → α-Al + Al_3_Sc). Firstly, Al_3_Zr can be precipitated directly from the melt when the content of Zr is high enough (>0.11 wt.%), Then, α-Al forms around the Al_3_Zr particle by the above peritectic reaction. Next, Al_3_Sc forms on the α-Al layer by the above eutectic reaction, and develops to the primary Al_3_(Sc_1−x_Zr_x_) phase [33,49,50]. It has been reported that impurities and the solidification cooling rate have great impacts on the formation of primary Al_3_(Sc_1−x_Zr_x_) phases. The impurities or microalloy elements such as Fe, Si, Ti, and Er are able to promote the heterogeneous nucleation (or the discontinuous precipitation) of primary Al_3_(Sc,Zr) or Al_3_(Sc,Zr, X) phases during solidification [33,49] while the rapid solidification cooling can effectively prevent the discontinuous precipitation and suppress the continuous precipitation [51]. In this work, due to the relatively low cooling rate during solidification, an excess of primary Al_3_(Sc_1−x_Zr_x_) phases developed and resulted in a fall of the corrosion resistance. The casting technology of rapid solidification will be a useful method to reduce the formation of primary Al_3_(Sc_1−x_Zr_x_) phases and the obvious corrosion resistance drop caused by the primary Al_3_(Sc_1−x_Zr_x_) phases is expected to be avoided in this way.

## 5. Conclusions

The composite addition of SC and Zr could bring about a remarkable grain refinement and effectively inhibit recrystallization. The strong effect of coherent Al_3_(Sc_1−x_Zr_x_) phases on the dislocation pinning resulted in the low recrystallization degrees in Al-Zn-Mg-Cu-Sc-Zr alloy, especially in the central layer of the extrusion material. The subgrain boundaries in extruded Al-Zn-Mg-Cu-Zr alloy can escape from the pinning of Al_3_Zr phases, and the subsequent merging of subgrains will result in obvious recrystallization either in the central layer or in the surface layer. The low recrystallization degree promotes GBPs precipitation with discontinuous distribution at the non-recrystallized grain boundaries, consequently bringing about high corrosion resistance in the central layer of Al-Zn-Mg-Cu-Sc-Zr alloy. The high content Sc addition contributes to the development of numerous primary Al_3_(Sc_1−x_Zr_x_) phases during solidification. These primary Al_3_(Sc_1−x_Zr_x_) phases will induce recrystallization under the particle-stimulated nucleation mechanism. Furthermore, the primary Al_3_(Sc_1−x_Zr_x_) phase can act as a cathode to stimulate an accelerated electrochemical process between the primary Al_3_(Sc_1−x_Zr_x_) particles and GBPs at the recrystallized grain boundaries, resulting in a dramatic decrease of corrosion resistance.

## Figures and Tables

**Table 1:** Chemical compositions of the researched alloys (in wt.%).

Alloys	Cu	Mg	Zn	Zr	Sc	Si	Fe	Al
Al-Zn-Mg-Cu-Zr	1.8	2.2	8.9	0.16	-	0.03	0.03	Bal.
Al-Zn-Mg-Cu-Sc-Zr	1.8	2.3	8.8	0.16	0.30	0.03	0.03	Bal.

**Table 2:** EDS analysis results of secondary phases (in at.%).

Spectrum Locations	Mg	Al	Cu	Zn	Sc	Zr
Spectrum 1	33.06	25.64	16.47	24.82	0	0
Spectrum 2	19.50	55.79	9.40	15.31	0	0
Spectrum 3	0	73.23	0	2.17	14.74	9.86

**Table 3:** Corrosion depths and widths at the representative IGC locations.

Samples	Depth/Width, μm/μm		
Center layer in Al-Zn-Mg-Cu-Zr alloy	287/875	145/2096	125/805
Center layer in Al-Zn-Mg-Cu-Sc-Zr alloy	117/742	82/524	75/751
Surface layer in Al-Zn-Mg-Cu-Zr alloy	166/626	108/893	85/1031
Surface layer in Al-Zn-Mg-Cu-Sc-Zr alloy	189/983	184/1222	175/1773

**Table 4:** Evaluated results of electrochemical parameters using Tafel slope analysis.

Samples	I_corr_/μA·cm^−2^	E_corr_/V_SCE_
Center layer in Al-Zn-Mg-Cu-Zr alloy	2.09	−0.796
Surface layer in Al-Zn-Mg-Cu-Zr alloy	2.33	−0.775
Center layer in Al-Zn-Mg-Cu-Sc-Zr alloy	1.50	−0.785
Surface layer in Al-Zn-Mg-Cu-Sc-Zr alloy	2.51	−0.783

**Table 5:** Electrochemical parameters obtained by fitting analysis of EIS.

Samples	Rs	Rt	CPE1-T	CPE1-P
Center layer in Al-Zn-Mg-Cu-Zr alloy	0.064	81.06	9.0036E-4	0.856
Surface layer in Al-Zn-Mg-Cu-Zr alloy	0.075	76.83	8.1234E-4	0.886
Center layer in Al-Zn-Mg-Cu-Sc-Zr alloy	0.063	97.29	8.4542E-4	0.876
Surface layer in Al-Zn-Mg-Cu-Sc-Zr alloy	0.105	57.19	1.5015E-4	0.886

## Data Availability

The data presented in this study are available on request from the corresponding author.
